# Genome-wide alternative polyadenylation dynamics underlying plant growth retardant-induced dwarfing of pomegranate

**DOI:** 10.3389/fpls.2023.1189456

**Published:** 2023-05-08

**Authors:** Xinhui Xia, Minhong Fan, Yuqi Liu, Xinyue Chang, Jingting Wang, Jingjing Qian, Yuchen Yang

**Affiliations:** ^1^ State Key Laboratory of Biocontrol, School of Ecology, Sun Yat-sen University, Shenzhen, China; ^2^ College of Agriculture, Anhui Science and Technology University, Fengyang, China

**Keywords:** alternative polyadenylation, plant growth retardant, pomegranate, post-transcriptional regulation, dwarfing

## Abstract

Dwarfed stature is a desired agronomic trait for pomegranate (*Punica granatum* L.), with its advantages such as lower cost and increased yield. A comprehensive understanding of regulatory mechanisms underlying the growth repression would provide a genetic foundation to molecular-assisted dwarfing cultivation of pomegranate. Our previous study induced dwarfed pomegranate seedlings *via* exogenous application of plant growth retardants (PGRs) and highlighted the important roles of differential expression of plant growth-related genes in eliciting the dwarfed phenotype of pomegranate. Alternative polyadenylation (APA) is an important post-transcriptional mechanism and has been demonstrated to act as a key regulator in plant growth and development. However, no attention has been paid to the role of APA in PGR-induced dwarfing in pomegranate. In this study, we characterized and compared APA-mediated regulation events underlying PGR-induced treatments and normal growth condition. Genome-wide alterations in the usage of poly(A) sites were elicited by PGR treatments, and these changes were involved in modulating the growth and development of pomegranate seedlings. Importantly, ample specificities were observed in APA dynamics among the different PGR treatments, which mirrors their distinct nature. Despite the asynchrony between APA events and differential gene expression, APA was found to regulate transcriptome *via* influencing microRNA (miRNA)-mediated mRNA cleavage or translation inhibition. A global preference for lengthening of 3’ untranslated regions (3’ UTRs) was observed under PGR treatments, which was likely to host more miRNA target sites in 3’ UTRs and thus suppress the expression of the corresponding genes, especially those associated with developmental growth, lateral root branching, and maintenance of shoot apical meristem. Together, these results highlighted the key role of APA-mediated regulations in fine-tuning the PGR-induced dwarfed stature of pomegranate, which provides new insights into the genetic basis underlying the growth and development of pomegranate.

## Introduction

Pomegranate (*Punica granatum* L.) is one type of the economic fruit trees that are widely cultivated across the globe. Because it is rich in vitamins and has antioxidant and anti-inflammatory properties in fruits, the health benefits of pomegranate are highly regarded, such as preventing or alleviating diseases and lowering high blood pressure or high cholesterol levels (National Center for Complementary and Integrative Health, NCCIH; Bourekoua et al., 2018; Shahamirian et al., 2019; Asrey et al., 2020; Turrini et al., 2020). With the fast-rising demand for pomegranate products, more and more attention has been paid to screen and breed pomegranate cultivars with the desired high fruit yield and quality. Dwarfing cultivation is one of the major focuses because of its advantages in plant photosynthetic efficiency, fruit production, and disease resistance compared to normal growing mode (Seleznyova et al., 2008; Foster et al., 2017; Wang et al., 2018; Zhou and Underhill, 2021). Qian et al. (2022) demonstrated that exogenous application of plant growth retardants (PGRs) can successfully elicit dwarfed pomegranate seedlings. Comparative transcriptome analysis further unraveled that PGR-mediated downregulation of plant growth hormone synthesis played a central role in inducing the dwarfed stature of pomegranate, providing new clues for molecular breeding of favorable dwarfed pomegranate varieties. Besides gene transcription, plant transcriptome is also under the regulation of post-transcriptional mechanisms, which have been demonstrated as a key contributor to the phenotypic plasticity of plants (Ye et al., 2019; Zhou et al., 2019; Singh and Roychoudhury, 2021). However, our current knowledge on the functional importance of post-transcriptional processes in pomegranate is still limited.

Polyadenylation [poly(A)] is an important post-transcriptional mechanism in eukaryotes that modulates mRNA maturation from the precursor mRNA (pre-mRNA). It includes two coupled steps: endonucleolytic cleavage at the 3’ end of pre-mRNA and the addition of a poly(A) tail at the cleavage sites (Colgan and Manley, 1997; Tian and Manley, 2017). More importantly, for many genes, the cleavage and poly(A) signal recognition occur at multiple positions, that is, giving rise to multiple mRNA isoforms with different lengths, which is referred to as alternative polyadenylation (APA). APA events have been demonstrated to be widespread across genomes; for example, over 70% of the *Arabidopsis* genes were found to possess more than one poly(A) site (Wu et al., 2011; Elkon et al., 2013). These APA events may alter the stability and translation of mRNA or the length of the resulting protein products; thus, APA serves as a key contributor to the complexity of eukaryotic transcriptome (Shen et al., 2008; Di Giammartino et al., 2011; Sun et al., 2012; Tian and Manley, 2017). Recent studies have highlighted the biological importance of APA in regulating plant growth, development, and resistance to environmental stresses (de Lorenzo et al., 2017; Zhou et al., 2019; Yu et al., 2022; Wang et al., 2023). For instance, Yu et al. (2022) performed a genome-wide investigation to APA dynamics underlying *Arabidopsis* leaf ontogeny and showed that the largest changes in poly(A) site usage occurred at the early stage of true leaf development, while the APA levels experienced a reduction along the developmental process. Furthermore, it was shown that these APA genes participated in modulating the biological processes associated with leaf development, for example, response to phytohormone. These findings highlighted the essential roles of APA-mediated post-transcriptional regulations in plant growth and development. However, the APA mechanisms underlying PGR-induced dwarfing have not been investigated in pomegranate.

In this study, we reanalyzed the published RNA-seq datasets (Qian et al., 2020) and characterized the genome-wide APA dynamics in the pomegranate seedlings treated with three kinds of PGRs, paclobutrazol, B9, and mannitol, to decipher the biological significance of APA-mediated mechanisms underlying PGR-induced dwarfing in pomegranate. Furthermore, we also compared the APA regulation to the gene expression changes, with the aim of dissecting the different contributions of transcriptional and post-transcriptional mechanisms to growth repression in pomegranate. Our findings will broaden our understanding of the genetic basis behind the PGR-elicited dwarfed stature of pomegranate and provide a foundation for future molecular-assisted dwarfing cultivation of pomegranate.

## Materials and methods

### Plant materials and data preprocessing

In our previous study, gene expression was characterized for the seedlings untreated (control group, CK) and treated with each of the three PGRs at different concentrations (paclobutrazol: 6 and 8 mg/L; B9: 6 and 8 mg/L; mannitol: 2.5 and 15 g/L) (Qian et al., 2022). Here, we reanalyzed the 14 RNA-seq datasets (two biological replicates for each scenario), which were deposited in the Gene Expression Omnibus (GEO) database of the National Center for Biotechnology Information (NCBI) under the accession number GSE195722, to investigate genome-wide poly(A) usage dynamics under the PGR treatments over CK. Data preprocessing was performed following the pipelines described in Qian et al. (2022). Briefly, for each dataset, low-quality bases, whose quality score < 20, and adapter contamination were first trimmed from the end of reads using Trim Galore (https://www.bioinformatics.babraham.ac.uk/projects/trim_galore/). Simultaneously, the reads with either error rate > 0.1 or ambiguous/N bases > 15 were discarded from the dataset. Finally, the sequences with length after trimming < 50 bp were also excluded from the downstream analysis. The clean reads were mapped to the pomegranate reference genome (the soft-seeded pomegranate cultivar “Tunisia”) *via* HISAT2 (Kim et al., 2019; Luo et al., 2020). The reads uniquely aligned to the genome were extracted and converted into bedgraph format using the sub-command *genomecov* of the BEDTools suite for downstream analysis (Quinlan and Hall, 2010).

### Differentially expressed alternative polyadenylation analysis

The gene model file for the reference genome in 12-column bed format (bed12) was converted from the GTF-format genome annotations file using the UCSC tools, *gtfToGenePred* and *genePredToBed* (https://genome.ucsc.edu/). The alignment result in bedgraph format and the gene model file were used as the inputs for the APA dynamics analysis using the APAtrap toolkit (Ye et al., 2018). Specifically, the annotated 3’ untranslated regions (3’ UTRs) were first refined and novel 3’ UTRs or 3’ UTR extensions were detected based on the mapping results of all the samples by the *identifyDistal3UTR* program. All the putative APA sites, as well as the usage level of APA sites, were predicted using *predictAPA* with default parameter settings. Differential usage analysis was performed for APA sites between CK and each of the treatment scenarios using the R package *deAPA*. The genes with an adjusted *p*-value < 0.05 and percentage difference (PD) ≥ 0.1 were considered to be significantly different in APA site usage between two groups, which were denoted as “differentially expressed APA genes (DAGs)”. The functional importance of the DAGs was assessed by Gene Ontology (GO) enrichment analysis using Fisher’s exact test, where the GO terms with *p*-value < 0.05 were considered to be significantly overrepresented compared to the genome background.

### Prediction of putative microRNA target sites

The majority of APA events occur in 3’ UTRs, that is, producing mRNA isoforms with 3’ UTRs of different lengths. Changes in the length of 3’ UTRs may cause the presence or loss of cis-regulatory elements, and thus pose influences on the stability, nuclear export, and translation efficiency of mRNA (Shen et al., 2008; Di Giammartino et al., 2011; Sun et al., 2012; Tian and Manley, 2017). Here, for each comparison, the DAGs were first grouped into two categories based on the Pearson product moment correlation coefficient *r*: (1) DAGs with *r* < 0 were supposed to contain more abundant proximal poly(A) site/shortened 3’ UTR under the treatment than CK, while (2) DAGs with *r* > 0 were indicated to use more distal poly(A) site/lengthened 3’ UTR in the treatment scenario. For each DAG of each category, the DNA sequence of each APA isoform was extracted from the reference genome using the sub-command *fastaFromBed* of BEDTools, and the putative microRNA (miRNA) target sites were identified in the 3’ UTR by screening against the collected miRNA sequences in miRBase (Release 21) using the psRNATarget web server (http://plantgrn.noble.org/psRNATarget/). The maximum cutoff of complementary matching score was set to 4.0. The isoforms undergoing 3’ UTR lengthening were supposed to be under the extra regulation of the miRNAs whose target sites were located in the lengthened 3’ end, compared to those with shorter 3’ UTRs.

### Comparison between differentially expressed alternative polyadenylation and differentially expressed genes

To further investigate the different regulatory roles of gene transcription and APA in PGR-induced dwarfing, we compared the DAGs to the differentially expressed genes (DEGs) detected in our previous study (Qian et al., 2022). The overlapping between DAGs and DEGs was visualized by a Venn diagram using the *draw.pairwise.venn* function of the R package VennDiagram. GO enrichment analysis was implemented with a cutoff *p*-value of 0.05 for the genes from each of the three categories: (1) the genes under the regulation of both differential expression and APA; (2) the genes specifically regulated by differentially expressed APA (DA-specific genes); and (3) the genes specifically regulated by differential expression (DE-specific genes).

## Results

### PGR-induced alternative polyadenylation changes play a substantial role in regulating pomegranate growth

**Figure 1:**
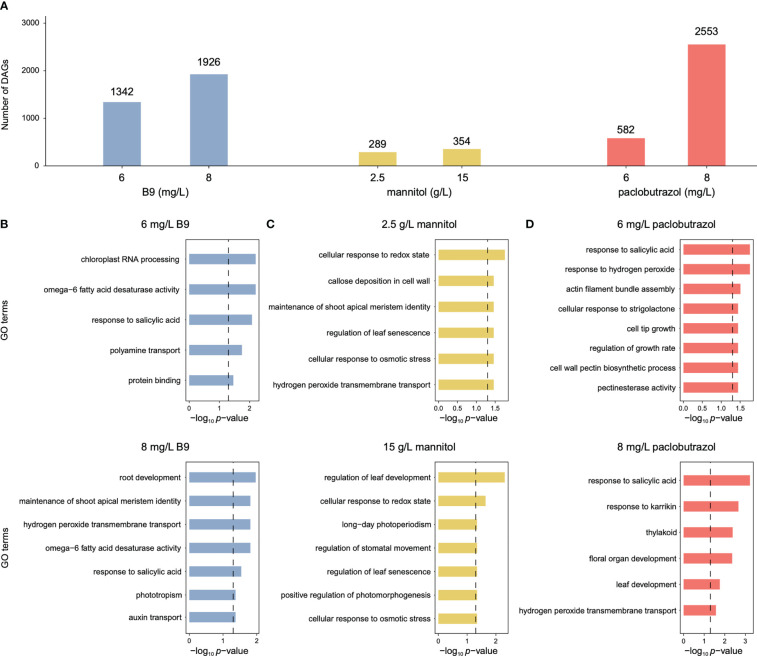
Numbers of DAGs induced by three PGRs and the corresponding enriched GO terms. **(A)** Numbers of DAGS identified under the treatment of B9 (blue), mannitol (yellow), and paclobutrazol (red). **(B–D)** Featur GO terms significantly enriched for the DAGs responsive to B9 **(B)**, mannitol **(C)**, and paclobutrazol **(D)**.

Compared to CK, exogenous applications of PGRs elicited 289–2,553 DAGs with significant differentiations in APA usage (**Figure 1A**). Functional enrichment analysis showed that these PGR-responsive APA events were associated with the biological processes of plant growth and development (**Figures 1B–D**). For instance, the DAGs induced by 8 mg/L B9 were enriched in auxin transport, root development, and maintenance of shoot apical meristem identity (**Figure 1B**), and mannitol-responsive DAGs were predominantly involved in the GO terms of leaf development and senescence, photomorphogenesis, stomatal movement, and cellular response to osmotic stress (**Figure 1C**). The application of 6 mg/L paclobutrazol was found to affect growth regulation and cell wall biosynthesis, and DAGs under the 8 mg/L treatment was overrepresented in leaf development (**Figure 1D**).

**Figure 2:**
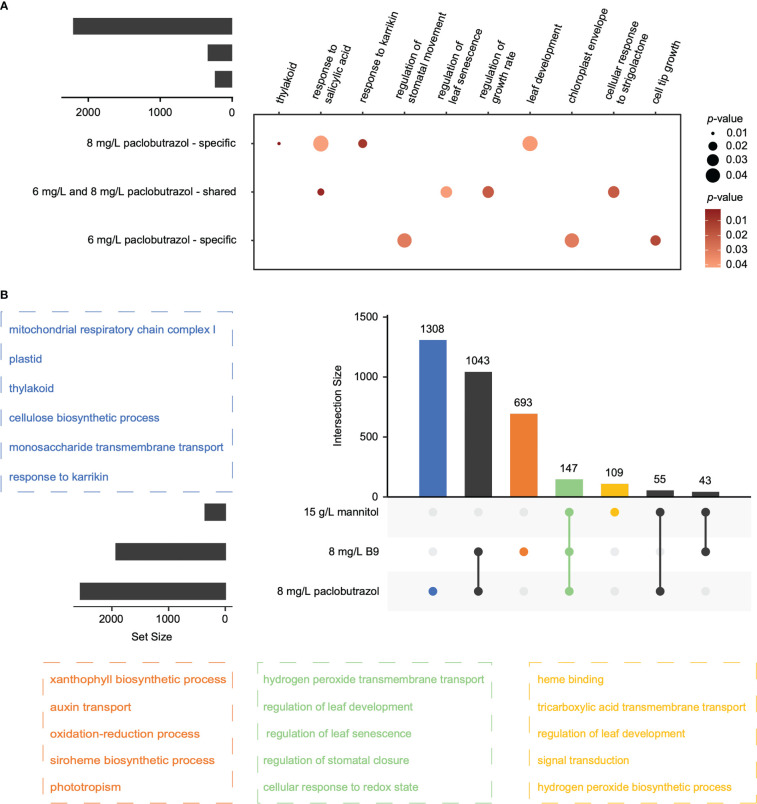
Overlap of the DAGs across treatments. **(A)** DAGs overlapped between 6 and 8 mg/L paclobutrazol treatments, and the representative GO terms enriched for each category. Circle size and color represent the significance level (*p*-value) of enrichment. **(B)** DAGs overlapped among the treatments of 8 mg/L paclobutrazol, 8 mg/L B9, and 15 g/L mannitol, and the representative GO terms enriched for the DAGs of different categories (highlighted by different colors).

For all the PGRs, the treatment at high concentration could provoke more alterations in APA profiles than that at low level (**Figure 1A**), which was consistent with their larger effects on suppressing the growth of pomegranate seedlings (Qian et al., 2022). With regard to paclobutrazol, 341 DAGs were shared between the treatments at the two concentrations, which were overrepresented in the regulations of growth rate and leaf senescence (**Figure 2A**). Comparatively, 241 and 2,212 genes displayed 3’ UTR alterations specifically under 6 and 8 mg/L treatment, respectively (**Figure 2A**). In particular, the DAGs specifically induced by 6 mg/L paclobutrazol were enriched in leaf development and thylakoid, whereas those responsive to 8 mg/L treatment were involved in cell tip growth and stomatal movement regulation (**Figure 2A**). Similar concentration-level specificities were also observed in the treatments of B9 and mannitol (**Supplementary Figure 1**). For instance, the 6 mg/L B9 treatment altered the poly(A) site usage of the genes related to seedling development, shoot apical meristem development, and cell wall thickening, and the DAGs identified under the 8 mg/L treatment were overrepresented in auxin transport, developmental process, and maintenance of shoot apical meristem identity (**Supplementary Figure 1B**, left panel). Together, these functional specificities of APA events unraveled the dose–response relationships of PGR treatments, which may assist to determine the optimal concentration for PGR application.

### Different alternative polyadenylation regulations were elicited by different PGRs

The APA changes also showed substantial specificities among different PGR treatments (**Figure 2B**). In total, 1,308, 693, and 109 APA events occurred exclusively when exogenously applied with 8 mg/L paclobutrazol, 8 mg/L B9, and 15 g/L mannitol, respectively, while only 147 events were observed in all these three treatments. Functional enrichment analysis showed that, the commonly changed events were supposed to mainly affect leaf development and senescence, stomatal closure, hydrogen peroxide transmembrane transport, and cellular response to redox state (**Figure 2B**). Comparatively, the DAGs specifically elicited by 8 mg/L paclobutrazol were enriched in the GO terms of mitochondrial respiratory chain complex I, thylakoid, cellulose biosynthesis, and karrikin response, while those exclusively occurring under the 8 mg/L B9 treatment were overrepresented in auxin transport, xanthophyll biosynthesis, and phototropism (**Figure 2B**). The biological processes involved in leaf development, tricarboxylic acid transmembrane transport, signal transduction, and hydrogen peroxide biosynthesis were enriched for the specially induced DAGs by 15 g/L mannitol (**Figure 2B**).

**Figure 3:**
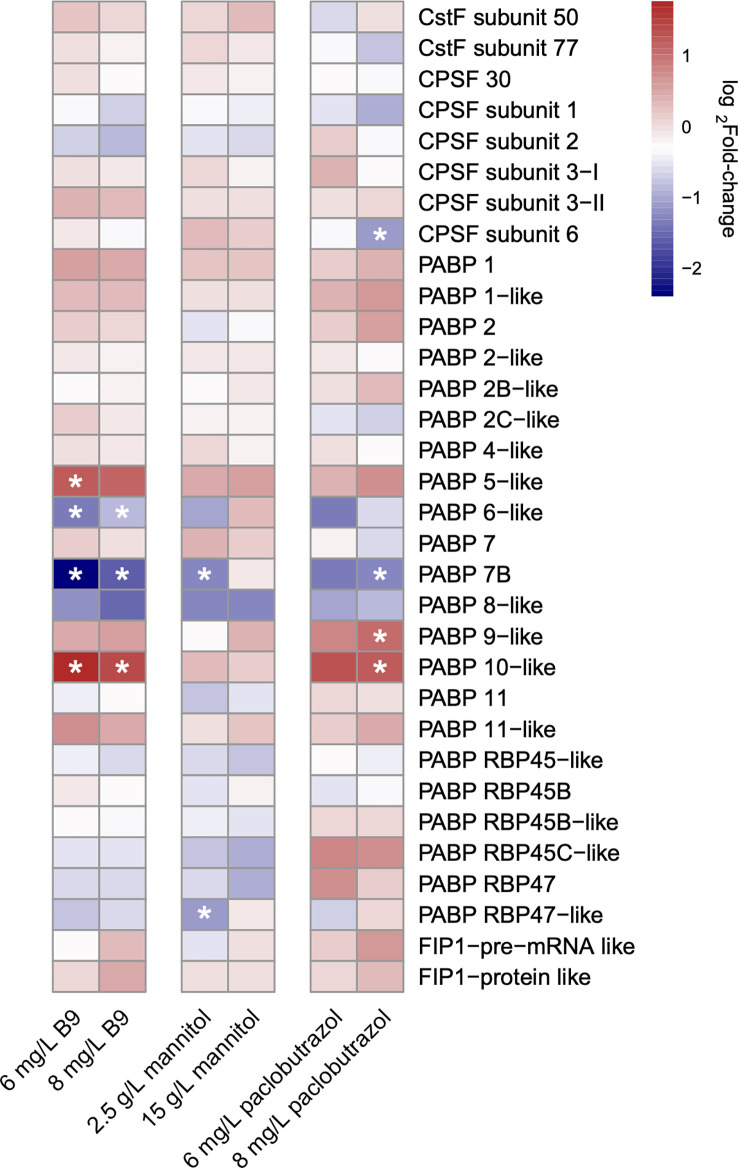
Heatmap illustrating the log2fold-change in the expression level of polyadenylation factor subunits under different treatments. * indicates the subunits of significantly differential expression under the treatment condition compared to CK.

Variations in the expression level of core polyadenylation factors, including polyadenylation machinery components, RNA-binding proteins, and transcription-related process, have been found to regulate APA (Hunt et al., 2012; Tian and Manley, 2017). Here, we first identified the genes encoding the subunits of four types of plant polyadenylation factors, cleavage stimulatory factor (CstF), cleavage and polyadenylation specificity factor (CPSF), poly(A) binding proteins (PABPs), and factor interacting with poly(A) polymerase (FIP1), in the pomegranate genome and compared their expression profiles under each treatment scenario to CK. The results showed that there were several polyadenylation factors significantly differentially expressed in response to the application of PGRs, which may play an important role in APA regulations (**Figure 3**). It is noteworthy that the expression profiles of polyadenylation apparatuses also displayed ample specificities among different treatments. Only one factor, PABP 7B, was commonly differentially expressed across all the three PGRs. In contrast, the expression of PABP 5 and 6 was specifically altered under the treatment of B9, and the gene that encodes PABP 9 and CPSF subunit 6 had an exclusively differential expression when treated with 8 mg/L paclobutrazol (**Figure 3**).

### Alternative polyadenylation and gene transcription variations play a relatively independent role in growth regulation

**Figure 4:**
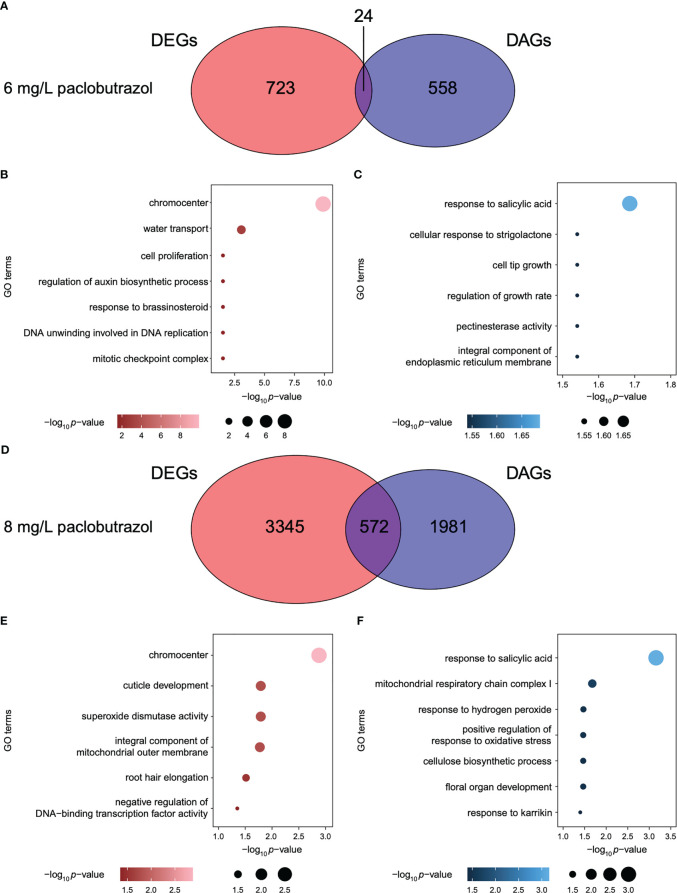
Comparison between DAGs and DEGs under the treatments of paclobutrazol. **(A)** Venn diagram illustrating the overlap between DAGs and DEGs when treated with 6 mg/L paclobutrazol. **(B, C)** GO terms enriched for the DA-specific **(B)** and DE-specific genes **(C)** under treatment of 6 mg/L paclobutrazol. **(D)** Venn diagram illustrating the overlap between DAGs and DEGs when treated with 8 mg/L paclobutrazol. **(E, F)** GO terms enriched for the DA-specific **(E)** and DE-specific genes **(F)** under the treatment of 8 mg/L paclobutrazol.

To explore the different roles of gene transcription and APA dynamics in the regulation of PGR-induced dwarfing in pomegranate, we compared DAGs identified in each treatment to DEGs of the corresponding scenario. When treated with PGRs, most genes were specifically under the regulation of either gene expression or APA (**Figure 4** and **Supplementary Figures 2, 3**). For instance, 24 and 572 out of the 582 and 2,553 DAGs, which accounted for 4.1% and 22.4%, were also differentially expressed under the treatment of 6 and 8 mg/L paclobutrazol, respectively (**Figures 4A, D**). The DE-specific genes induced by 6 mg/L paclobutrazol were highly represented in cell proliferation, auxin biosynthesis, and brassinosteroid (BR) response (**Figure 4B**), while the DA-specific genes were predominantly involved in growth regulation, pectinesterase activity, and responses to salicylic acid and strigolactone (**Figure 4C**). When exposed to 8 mg/L paclobutrazol, genes related to superoxide dismutase activity and root hair elongation were likely to be exclusively differentially expressed compared to CK (**Figure 4E**), whereas those participating in cellulose biosynthesis, oxidative stress regulation, and responses to salicylic acid and karrikin showed different APA usages (**Figure 4F**).

A similar pattern was also observed under the treatment of B9 and mannitol: only 4.2%–14.2% of DAGs were overlapped with DEGs (**Supplementary Figures 2A, E** and **3A, E**). When treated with 8 mg/L B9, for one example, the DA-specific genes were significantly enriched in the processes of root development, maintenance of shoot apical meristem identity, and auxin transport (**Supplementary Figure 2F**), while the DE-specific genes were overrepresented in cell wall catabolism and oxidative stress responses (**Supplementary Figure 2G**). With regard to the application of 2.5 g/L mannitol, the DA-specific genes were highly represented in cellular response to osmotic stress, callose deposition in cell wall, and leaf senescence (**Supplementary Figure 3B**), and the GO terms related to cell proliferation, growth, and development were found to be enriched for the genes specifically regulated by differential expression (**Supplementary Figure 3C**). Comparatively, when treated with 15 g/L mannitol, the light-mediated leaf development, leaf senescence, and photoperiodism were mainly regulated by APA events (**Supplementary Figure 3F**), whereas the genes involved in auxin metabolism, cell development-related programmed cell death, cell wall thickening, secondary shoot formation, superoxide radical removal, and L-ascorbic acid transmembrane transport were largely under the control of different expression (**Supplementary Figure 3G**). Taken together, these results suggested that, in many scenarios, APA and gene transcription regulate different aspects of the growth and development of pomegranate seedlings and together contribute to the PGR-induced dwarfed stature.

### Changes in 3’ UTR length affect microRNA target sites

**Figure 5:**
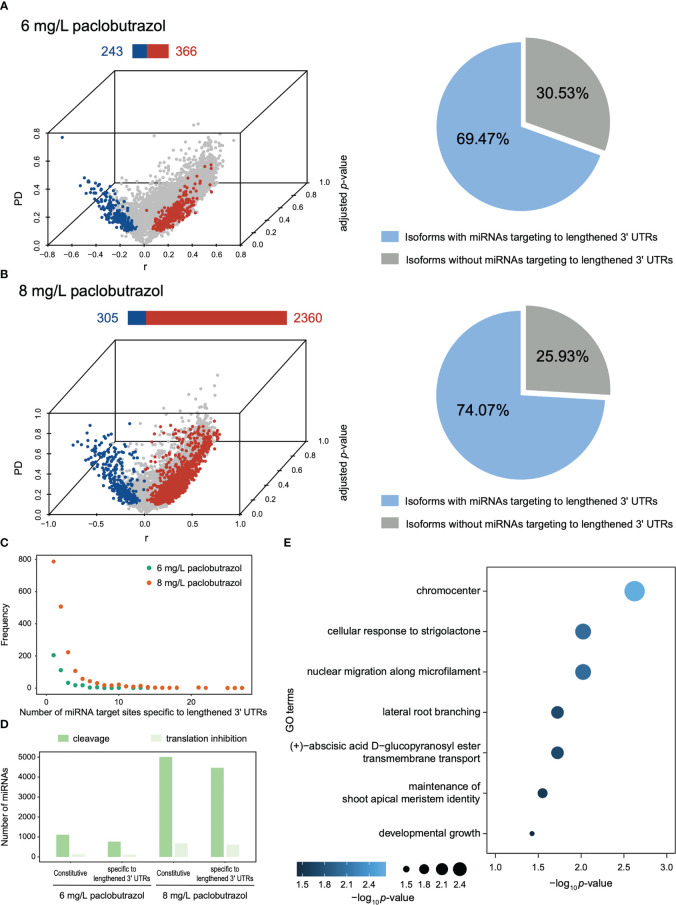
Overview of DAGs with lengthened/shortened 3’ UTR and the putative miRNAs targeting to the lengthened 3’ UTRs under the treatment of paclobutrazol. **(A, B)** Left panels: 3D volcano plot illustrating the DAGs displaying 3’ UTR lengthening (red) and shortening (blue) when treated with 6 **(A)** and 8 mg/L paclobutrazol **(B)**. The bar above each volcano plot shows the number of DAGs using more longer (red) and shorter 3’ UTRs (blue). Right panels: Pie charts showing the proportion of isoforms with (blue) or without (gray) putative miRNAs targeting the lengthened area of 3’ UTRs under 6 mg/L **(A)** and 8 mg/L paclobutrazol treatment **(B)**. **(C)** Frequency distribution illustrating the number of miRNA target sites identified specifically in lengthened 3’ UTRs across genes, when treated with 6 mg/L (green) and 8 mg/L paclobutrazol (orange), respectively. **(D)** Numbers of constitutive (existing in isoforms with both short and long 3’ UTRs) or lengthened 3’ UTR-specific miRNAs that were predicted with putative mRNA cleavage (green) and translation inhibition potentials (light green) under two paclobutrazol treatment scenarios, respectively. **(E)** Representative GO terms enriched for the DAGs that were with miRNAs targeting the lengthened 3’ UTR area and significantly downregulated in response to 8 mg/L paclobutrazol treatment.

APA events were also found to substantially modulate gene expression at post-transcriptional and translational levels. Compared to CK, DAGs in the PGR-treated seedlings displayed a global preference for using distal poly(A) sites (**Figures 5A, B** and **Supplementary Figures 4A**, **5A**). For example, under the treatment of 8 mg/L paclobutrazol, 2,360 DAGs exhibited a higher abundance of the isoforms with longer 3’ UTRs, while only 305 genes used more proximal poly(A) sites (**Figure 5B**). These lengthened 3’ UTRs were supposed to host more miRNA target sites, which can further modulate mRNA abundance by influencing their stability. Consistently, 65.99%–74.07% of the isoforms using longer 3’ UTRs were inferred to consist of extra miRNA target sites, compared to those using shorter ones, under all the treatment scenarios (**Figures 5A, B** and **Supplementary Figures 4B**, **5B**). For up to 61 isoforms, more than 10 putative miRNA targets were under the impact of the changes in 3’ UTR length (**Figure 5C** and **Supplementary Figures 4C, 5C**). Most of these miRNAs, both constitutive (existing in isoforms with both short and long 3’ UTRs) and lengthened 3’ UTR-specific miRNAs, were identified to function in cleavage of the corresponding mRNA, while 9.00%–12.73% of the miRNAs specific to the extended 3’ UTRs were supposed to inhibit mRNA translation (**Figure 5D** and **Supplementary Figures 4D**, **5D**). More important, up to 259 DAGs with lengthened 3’ UTRs targeted by miRNAs were significantly downregulated under the treatment of PGRs (**Supplementary Figures 4E, 5E, 6**). When treated with 8 mg/L paclobutrazol, the miRNA-mediated downregulated genes were overrepresented in the GO terms of developmental growth, lateral root branching, maintenance of shoot apical meristem identity, and cellular response to strigolactone, indicative of their important roles in regulating the growth and development of pomegranate seedlings (**Figure 5E**).

## Discussion

In the current study, we explored the PGR-induced APA dynamics using the RNA-seq data from our previous study and showed that all the PGR treatments, even at low concentrations, provoked genome-wide alterations in the usages of poly(A) sites (**Figure 1A**). These changes were found to substantially influence how pomegranate seedlings grew and developed. For example, the poly(A) site usages of the genes involved in auxin transport and growth regulation were significantly altered after the treatments (**Figures 1B–D**). In particular, one DAG encodes protein PIN-LIKES, which functions as an efflux carrier that mediates the unidirectional auxin flow (Polar auxin transport, PAT) among plant tissues (Křeček et al., 2009). The gene encoding mitogen-activated protein kinase kinase 2 (MKK2) also displayed significantly different APA profiles under the treatment. MKK2, together with mitogen-activated protein kinase 10 (MPK10), forms a module of mitogen-activated protein kinase (MAPK) signaling pathways that serves as a key regulator for PAT in plants (Jagodzik et al., 2018). These alterations in auxin transport may make contributions to the repressed growth and development in pomegranate. Correspondingly, the APA usages of the genes involved in shoot apical meristem identity maintenance and leaf development were also changed in response to PGR treatments (**Figures 1**, **2B**). One of such genes is the calpain-type cysteine protease encoding gene *DEK1*. Studies in *Physcomitrella patens* highlighted the important function of *DEK1* in controlling the cell fate transition from 2D to 3D growth, where *DEK1* knockout in *P. patens* led to aberrant cell divisions and developmental arrest in buds (Demko et al., 2014; Johansen et al., 2016). Together, these widespread alterations in APA profiles under PGR treatments indicate the substantial significance of post-transcriptional mechanisms in modulating the dwarfed stature of pomegranate.

The APA dynamics display ample specificities among the treatments of different types/concentrations of PGRs (**Figures 1**, **2**), which correspond to their distinct nature. The DAGs induced by 8 mg/L paclobutrazol were particularly associated with the response for karrikins, a type of plant growth regulator that controls plant development (Wang et al., 2020), while the genes with significant APA changes under 8 mg/L B9 treatment were overrepresented in phototropism and xanthophyll biosynthesis (**Figure 2B**). It is consistent with our previous observations from the transcriptome data that genes responsive to strigolactones, a type of plant signaling compound with similar biochemical properties and physiological activities to karrikins, were specifically downregulated when exposed to 8 mg/L paclobutrazol, whereas those involved in photosynthesis and photosystem II assembly/repair were suppressed by the application of B9 (Qian et al., 2022). Compared to B9 and paclobutrazol, mannitol treatments at both concentrations were found to elicit cellular response to osmotic stress (**Figure 1C**), corresponding to its specific mechanism that mannitol represses plant growth and development by increasing ambient osmatic pressure and causing drought stress to plants (Bhat and Chandel, 1993); thus, antioxidant reactions were activated to alleviate the oxidative damage. These results indicated that different regulatory mechanisms underlying the pomegranate dwarfing elicited by different PGRs were employed.

In the current study, we found that, in most scenarios, APA and transcriptional regulations are not synchronized and modulate PGR-induced growth repression *via* different routes, as manifested by both the little overlap between DAGs and DEGs and the differences in the pathways modulated by DAGs and DEGs (**Figures 4A, D** and **Supplementary Figures 2A, E** and **3A, E**). The transcriptome data revealed that paclobutrazol obviously downregulated the genes related to the tryptophan-independent auxin biosynthetic process (Qian et al., 2022). Comparatively, APA events predominantly affected the polar transport of auxin among tissues. Similarly, when treated with 2.5 g/L mannitol, the cell wall modification process was modulated by the changes in both gene transcription and APA, although in distinct ways (**Supplementary Figure 3**). In particular, the gene encoding endoglucanase 8 (CEL1), which is a type of cellulose-hydrolyzing enzyme that regulates the cell wall relaxation associated with cell growth and expansion, was significantly downregulated (Tsabary et al., 2003; Shani et al., 2006). Consequently, the suppression of CEL1 would disrupt the differentiations of the plant vascular system and lead to shorter roots and shoots (Tsabary et al., 2003). In contrast, UTP-glucose-1-phosphate uridylyltransferase (also referred to as UDP-glucose pyrophosphorylase, UGPase), which supplies UDP-glucose substrate for the formation of secondary cell wall in plants, displayed substantial poly(A) usage variations. Moreover, Payyavula et al. (2014) showed that the maintenance of UGPase’s function was important for the normal growth of *Populus deltoides*. These results suggested that both APA and gene transcription make key contributions to the intricate regulatory network underlying the dwarfing stature of pomegranate.

Despite the independent function of APA in regulation, APA is able to modulate transcriptome *via* influencing the presence or absence of regulatory elements located in 3’ UTRs. Here, the DAGs induced by PGR treatments, especially at high concentrations, displayed a global preference for 3’ UTR lengthening (**Figures 5A, B** left panel and **Supplementary Figures 4A, 5A**), which anchor more miRNA target sites than the corresponding shorter 3’ UTRs (**Figures 5A, B** right panel and **Supplementary Figures 4B, 5B**). The majority of these extra “burdens” were supposed to suppress gene expression by causing the cleavage or destabilization of mRNA (**Figure 5D** and **Supplementary Figures 4D, 5D**). In particular, 259 DAGs with miRNAs specifically targeted in the lengthened 3’ UTRs were significantly downregulated in response to the 8 mg/L paclobutrazol treatment (**Supplementary Figure 6**). These genes were found to participate in developmental growth, lateral root branching, and maintenance of shoot apical meristem (**Figure 5E**). Of them, the gene encoding E3 ubiquitin-protein ligase (KEG) is known by its negative regulatory activity of abscisic acid (ABA) signaling, and the suppression of its expression has been shown to retard the growth of *A. thaliana* (Stone et al., 2006). As another example, the expression of the gene that encodes ammonium transporter 1 member 1 (AMT1;1) was also supposed to be suppressed. AMT1;1 plays an important role in ammonium uptake from soil solution by roots and the subsequent root-to-shoot transport of ammonium; thus, the inhibition of AMT1;1 would lead to nitrogen deficiency and growth defect in pomegranate seedlings (Mayer and Ludewig, 2006). Together, these results highlight the role of APA events in fine-tuning gene expression in response to PGR treatments, which makes a key contribution to the retarded growth and development in the dwarfed pomegranate seedlings.

## Conclusion

In this study, we, for the first time, identified and characterized the APA dynamics underlying PGR-elicited dwarfing in pomegranate. Our findings highlight the biological importance of post-transcriptional mechanisms in modulating pomegranate growth and development, which adds a new dimension to the genetic basis of the agronomic trait of pomegranate. However, since our study is mainly based on the prediction from RNA-seq, we might be lacking in power to capture all of the signals and miss some of the true APA events. Thus, in the future, a more comprehensive investigation on the poly(A) usage alterations in pomegranate is essential using efficient technology to measure 3’ UTR dynamics, such as Poly(A) tag sequencing (PAT-seq).

## Data availability statement

The datasets we used in this study can be found in online repositories National Center for Biotechnology Information (NCBI) Gene Expression Omnibus (GEO) with accession number of GSE195722.

## Author contributions

YY and JQ designed the study. XX, MF, and YY collected the data and performed the bioinformatic analyses. XX, YL, XC, JW, JQ, and YY wrote the manuscript. All authors contributed to the article and approved the submitted version.
