# Supplementary material for: Genome-wide alternative polyadenylation dynamics underlying plant growth retardant-induced dwarfing of pomegranate
Source: Front Plant Sci. 2023 May 8;14:1189456. doi: 10.3389/fpls.2023.1189456 (PMC10200943; doi:10.3389/fpls.2023.1189456)
Supplement: Supplementary file 1 [file DataSheet_1.docx]

**Supplementary materials for Online Publication**

**Genome-wide Alternative Polyadenylation Dynamics Underlying Plant Growth Retardant-induced Dwarfing of Pomegranate**

Xinhui Xia^1^, Minhong Fan^1^, Yuqi Liu^1^, Xinyue Chang^1^, Jingting Wang^2^, Jingjing Qian^2*^, Yuchen Yang^1*^

1 State Key Laboratory of Biocontrol, School of Ecology, Sun Yat-sen University, Guangzhou, China, 2 College of Agriculture, Anhui Science and Technology University, Fengyang, China.

^*^ Corresponding author

**
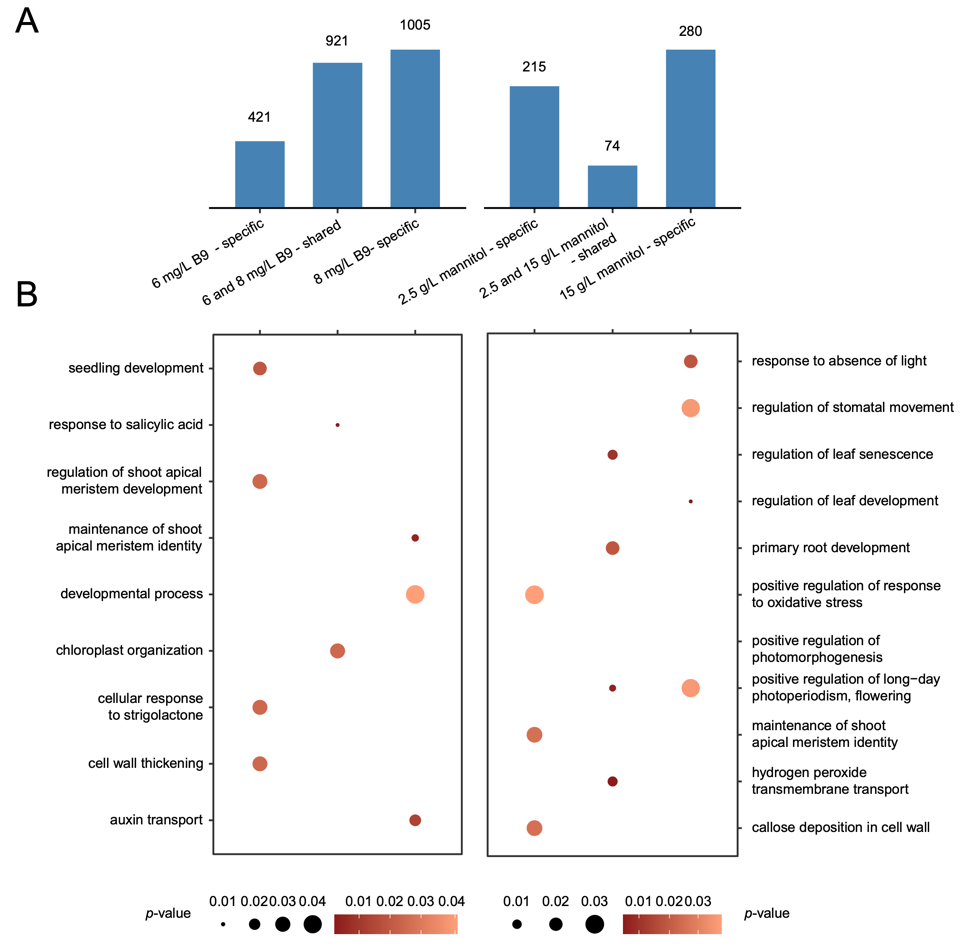
**

**Supplementary Figure 1.** Comparison of the DAGs responsive to different PGR concentrations. (**A**) Overlap of the DAGs under the treatments of 6 and 8 mg/L B9 (left panel) and the treatments of 2.5 and 15 g/L mannitol (right panel). (**B**) Featured GO terms enriched for the DAGs in each category of B9 treatment (left panel) and mannitol (right panel). Circle size and color represent the significance level (*p*-value) of enrichment.

**
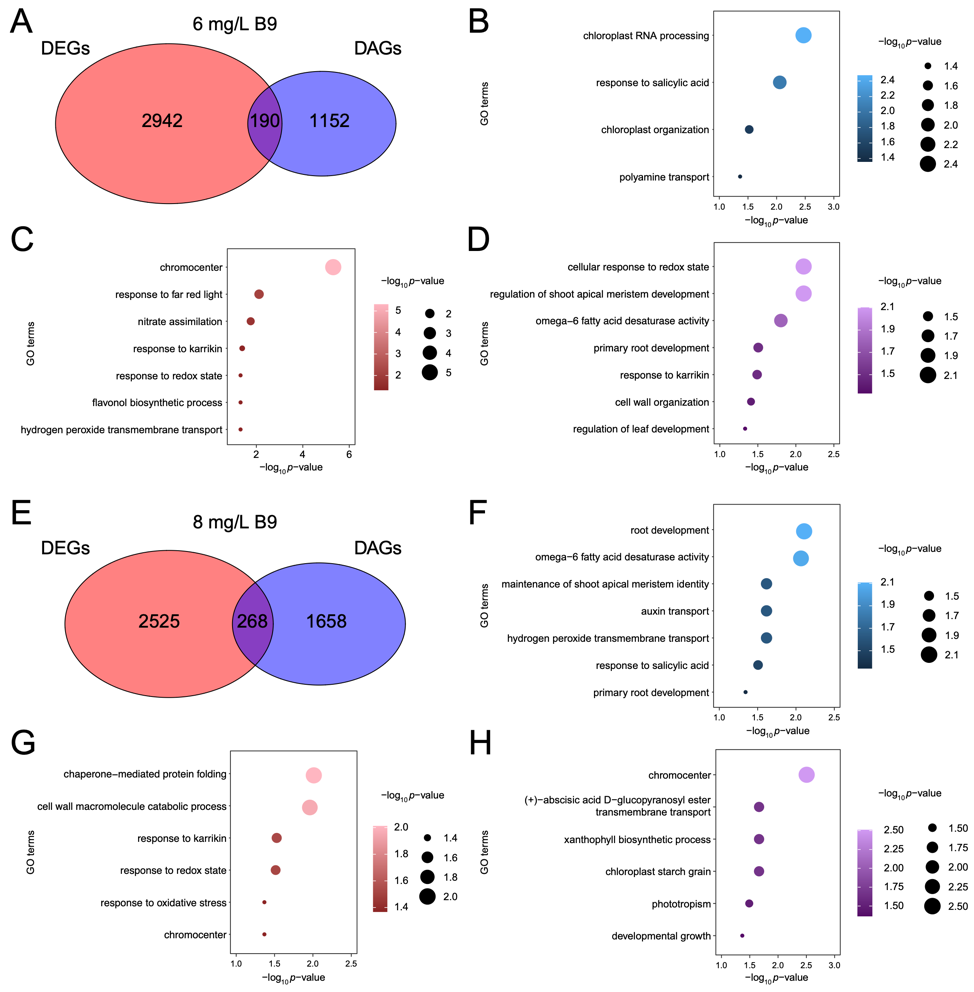
**

**Supplementary Figure 2.** Comparison between DAGs and DEGs under the treatments of 6 and 8 mg/L B9. (**A**) Venn diagram illustrating the overlap between DAGs and DEGs when treated with 6 mg/L B9. (**B-D**) GO terms enriched for the DA-specific genes (**B**), DE-specific genes (**C**) and genes regulated by both differential expression and APA (**D**), under treatment of 6 mg/L B9. (**E**) Venn diagram illustrating the overlap between DAGs and DEGs when treated with 8 mg/L B9. (**F-H**) GO terms enriched for the DA-specific genes (**F**), DE-specific genes (**G**) and the genes regulated by both differential expression and APA (**H**), under the treatment of 8 mg/L B9.

**
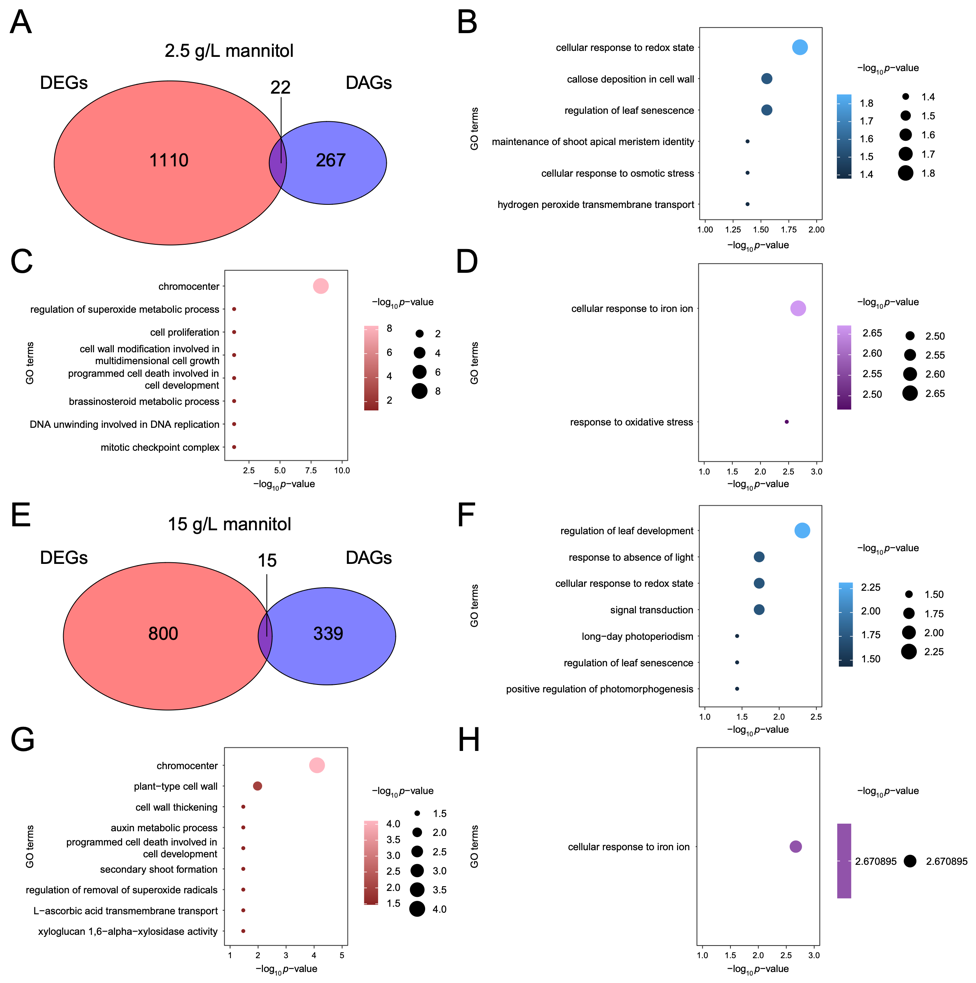
**

**Supplementary Figure 3.** Comparison between DAGs and DEGs under the treatments of 2.5 and 15 g/L mannitol. (**A**) Venn diagram illustrating the overlap between DAGs and DEGs when treated with 2.5 g/L mannitol. (**B-D**) GO terms enriched for the DA-specific genes (**B**), DE-specific genes (**C**) and genes regulated by both differential expression and APA (**D**), under the treatment of 2.5 g/L mannitol. (**E**) Venn diagram illustrating the overlap between DAGs and DEGs when treated with 15 g/L mannitol. (**F-H**) GO terms enriched for the DA-specific genes (**F**), DE-specific genes (**G**) and genes regulated by both differential expression and APA (**H**), under the treatment of 15 g/L mannitol.


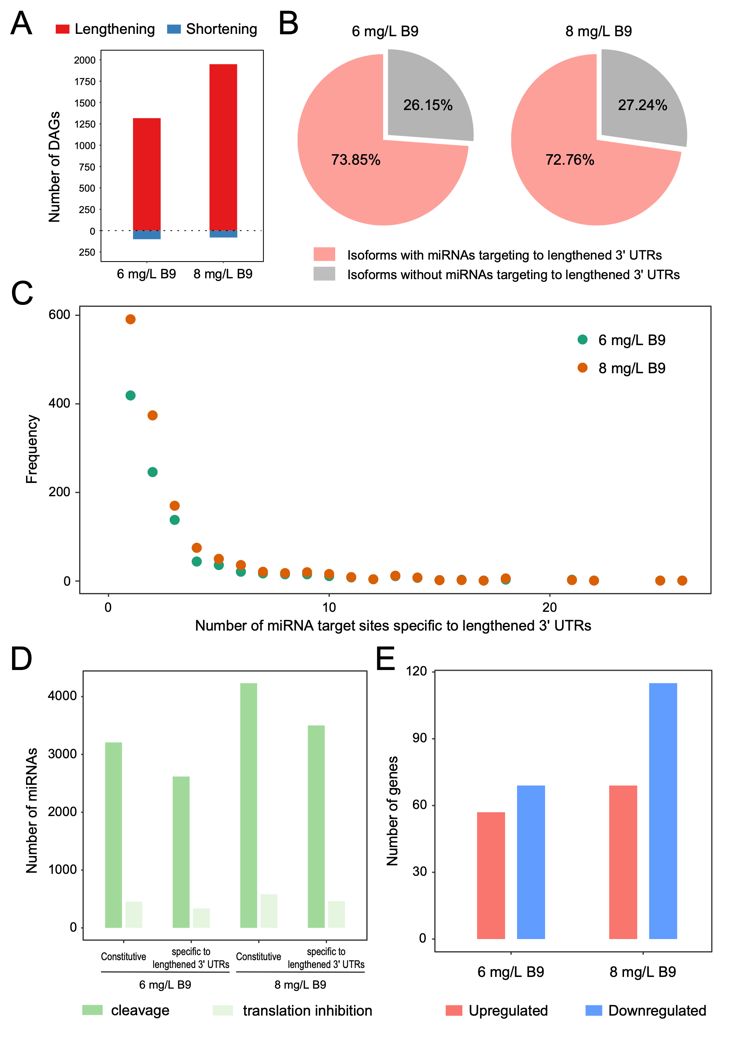


**Supplementary Figure 4**. Overview of miRNA target sites identified in the 3' UTRs of the DAGs under the treatment of 6 and 8 mg/L B9. (**A**) The number of DAGs displaying 3' UTR lengthening (red) and shortening (blue) when treated with B9. (**B**) Proportion of isoforms with (red) or without (grey) miRNA target sites located in lengthened 3' UTRs. (**C**) Frequency distribution illustrating the number of miRNA target sites identified specifically in lengthened 3' UTRs across genes, when treated with 6 (green) and 8 mg/L B9 (orange), respectively. (**D**) Numbers of constitutive (existing in isoforms with both short and long 3' UTRs) or lengthened 3' UTR-specific miRNAs that were predicted with putative mRNA cleavage (green) and translation inhibition potentials (light green) under two B9 treatment scenarios, respectively. (**E**) Number of DAGs with miRNA target sites located specifically in lengthened 3' UTRs and significantly up- (red) and downregulated (blue) under the treatments of 6 and 8 mg/L B9.


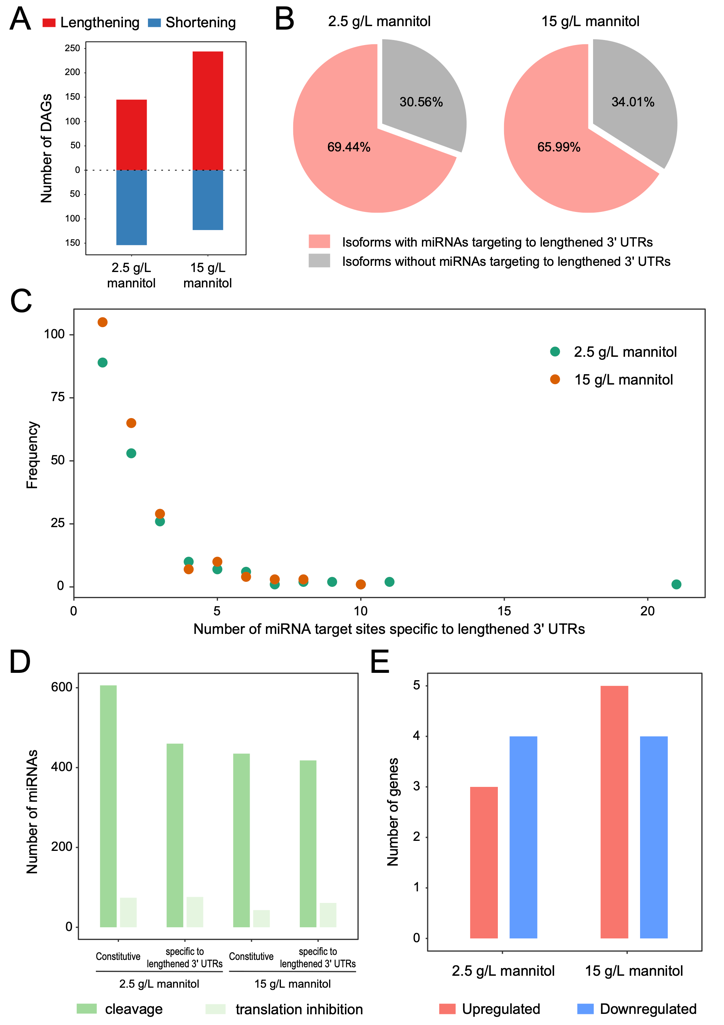


**Supplementary Figure 5.** Overview of miRNA target sites identified in the 3' UTRs of the DAGs under the treatment of 2.5 and 15 g/L mannitol. **(A)** The number of DAGs displaying 3' UTR lengthening (red) and shortening (blue) when treated with mannitol. (**B**) Proportion of isoforms with (red) or without (grey) miRNA target sites located in lengthened 3' UTRs. (**C**) Frequency distribution illustrating the number of miRNA target sites identified specifically in lengthened 3' UTRs across genes, when treated with 2.5 (green) and 15 g/L mannitol (orange), respectively. (**D**) Numbers of constitutive (existing in isoforms with both short and long 3' UTRs) or lengthened 3' UTR-specific miRNAs predicted with mRNA cleavage (green) and translation inhibition potentials (light green) under two mannitol treatments, respectively. (**E**) Number of DAGs with miRNA target sites located specifically in lengthened 3' UTRs and significantly up- (red) and downregulated (blue) under the treatments of 2.5 and 15 g/L mannitol.


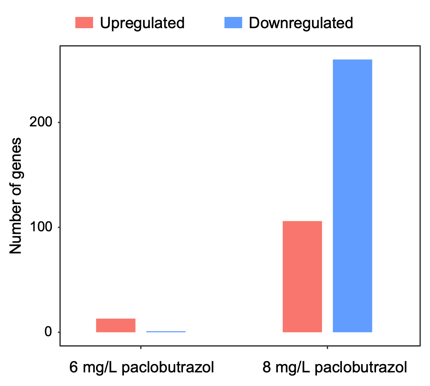


**Supplementary Figure 6.** Number of DAGs with miRNA target sites located specifically in lengthened 3ʹ UTRs and significantly up- and downregulated under the treatments of 6 and 8 mg/L paclobutrazol.
